# ECG challenge: ST-segment elevation after pulsed-field ablation

**DOI:** 10.1093/ehjcr/ytag483

**Published:** 2026-06-26

**Authors:** Thai Duy Vo, Ngoc Dung Kieu, Chin-Yu Lin

**Affiliations:** Division of Cardiac Electrophysiology, Cardiovascular Center, Taipei Veterans General Hospital, No. 201, Sec. 2, Shipai Rd., Beitou District, Taipei 11217, Taiwan; Department of Arrhythmology, Cho Ray Hospital, 201b Nguyen Chi Thanh, Cho Lon Ward, Ho Chi Minh city, Vietnam; Department of Arrhythmology, Cho Ray Hospital, 201b Nguyen Chi Thanh, Cho Lon Ward, Ho Chi Minh city, Vietnam; Division of Cardiac Electrophysiology, Cardiovascular Center, Taipei Veterans General Hospital, No. 201, Sec. 2, Shipai Rd., Beitou District, Taipei 11217, Taiwan; Department of Medicine, National Yang-Ming Chiao-Tung University, Taipei 112304, Taiwan

**Keywords:** ST elevation, Pulsed field ablation, Atrial fibrillation, Electrocardiographic artifact, Coronary vasospasm

## Clinical vignette

An 85-year-old woman with hypertension, type 2 diabetes mellitus, prior hemicolectomy for ascending colon adenocarcinoma, and end-stage kidney disease on maintenance haemodialysis underwent pulsed-field ablation for symptomatic paroxysmal atrial fibrillation, with concomitant left atrial appendage occlusion. Pulmonary vein isolation and occluder deployment were completed without immediate complication under fluoroscopic and electroanatomic guidance (see Supplementary material online, *Figure S2*). One hour later, she developed acute dyspnoea and severe hypertension (blood pressure 210/130 mmHg). She reported no typical chest pain; however, her renal failure, advanced age, and recent left atrial instrumentation made silent peri-procedural myocardial infarction an important concern. A 12-lead ECG was obtained (*Figure 1*). It showed an intermittent junctional rhythm at 51 b.p.m., apparent ST-segment elevation in V1–V4, and broad, hyperacute T waves in II, III, and aVF. No single coronary territory explained all the abnormalities, as the limb and precordial leads were involved in a non-contiguous pattern. The differential diagnosis included acute coronary occlusion, coronary air embolism, pulsed-field ablation-related coronary vasospasm, a hypertensive repolarisation abnormality, hyperkalaemia, and technical artefact.

**Figure 1:**
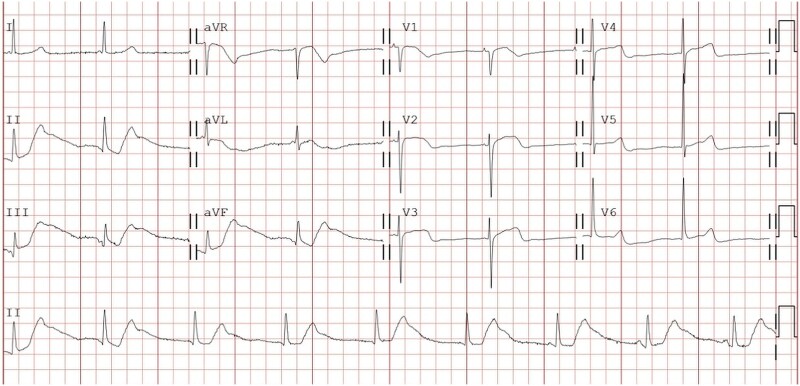
Twelve-lead electrocardiogram obtained 1 h after pulsed-field ablation and left atrial appendage occlusion, showing an intermittent junctional rhythm, apparent ST-segment elevation in V1–V4, and broad, hyperacute T waves in the inferior leads.

## Question 1

What is the most likely explanation for these ECG findings?

- Acute anteroseptal ST-elevation myocardial infarction due to left anterior descending coronary artery occlusion
- Pulsed-field ablation-related coronary vasospasm as the sole mechanism
- Arterial pulse-tapping (electrode-motion) artefact mimicking ST-elevation myocardial infarction
- Hyperkalaemia with a pseudoinfarction pattern
- Acute pericarditis after left atrial instrumentation

**Correct answer:** C

**Explanation:** Although the tracing appeared alarming, the pattern was anatomically discordant: the limb and precordial leads were distorted without a coherent single-vessel distribution. Arterial pulse-tapping artefact arises when an electrode overlies, or is mechanically coupled to, a pulsatile artery or vascular access; cardiac-cycle-synchronous motion can produce apparent ST-segment elevation and pseudo-hyperacute T waves. Here, complete normalization after electrode repositioning strongly favoured this technical mimic.^1^

## Question 2

Which bedside step is most useful before committing a haemodynamically stable patient to irreversible reperfusion therapy?

- Administer fibrinolysis immediately because any anterior ST elevation after ablation is STEMI
- Repeat the ECG after confirming calibration and moving electrodes away from arterial pulsation or arteriovenous access
- Treat empirically for pericarditis with high-dose non-steroidal anti-inflammatory drugs
- Give intravenous calcium gluconate without checking electrolytes
- Ignore the tracing because ST elevation after ablation is expected

**Correct answer:** B

**Explanation:** Post-procedural ST-segment elevation should be regarded as potentially ischaemic until promptly reassessed. In a stable patient, however, an immediate ECG audit is low-risk and high-yield: verify lead identity, calibration, and electrode contact, then repeat the tracing after moving the electrodes away from pulsatile sources. In this case, the repeat ECG after blood pressure control and electrode repositioning normalized, avoiding unnecessary invasive escalation.^1,2^

## Question 3

What is the safest management strategy after the tracing normalizes?

- Discharge the patient without further observation
- Proceed directly to routine coronary angiography in all patients after pulsed-field ablation
- Continue close monitoring with serial ECGs and biomarkers, treat severe hypertension, consider bedside echocardiography, and reserve urgent coronary evaluation for persistent or recurrent ischaemic evidence
- Start dual antiplatelet therapy and anticoagulation solely because the first ECG showed ST elevation
- Schedule elective outpatient ECG follow-up only

**Correct answer:** C

**Explanation:** Normalization after lead correction greatly lowers the likelihood of sustained transmural infarction but does not end the assessment. The immediate post-ablation period still warrants vigilance for coronary vasospasm, air embolism, thromboembolism, and hypertensive myocardial stress. Persistent ST-segment elevation, recurrent symptoms, haemodynamic compromise, a new regional wall-motion abnormality, arrhythmia, or a diagnostic troponin rise should prompt urgent coronary evaluation.^2–4^

## Supplementary Material

ytag483_Supplementary_Data

## Data Availability

The data that support the findings of this study are available from the corresponding author upon request.

## Supplementary material

Supplementary material online, *Figure S1* shows the repeat ECG after intravenous nicardipine and electrode repositioning away from arterial pulsation, with resolution of the ST-segment elevation and normalization of the inferior T waves. Supplementary material online, *Figure S2* shows the procedural fluoroscopic and electroanatomic imaging.
