# Supplementary material for: ECG challenge: ST-segment elevation after pulsed-field ablation
Source: Eur Heart J Case Rep. 2026 Jun 26;10(7):ytag483. doi: 10.1093/ehjcr/ytag483 (PMC13332503; doi:10.1093/ehjcr/ytag483)
Supplement: ytag483_Supplementary_Data [file ytag483_supplementary_data.zip › Supplementary_Legends.docx]

**Supplementary material**

Supplementary Figure S1 shows the repeat ECG after intravenous nicardipine and electrode repositioning away from arterial pulsation, with resolution of the ST-segment elevation and normalisation of the inferior T waves. Supplementary Figure S2 shows the procedural fluoroscopic and electroanatomic imaging.
