# Supplementary figures and images for: ECG challenge: ST-segment elevation after pulsed-field ablation
Source: Eur Heart J Case Rep. 2026 Jun 26;10(7):ytag483. doi: 10.1093/ehjcr/ytag483 (PMC13332503; doi:10.1093/ehjcr/ytag483)

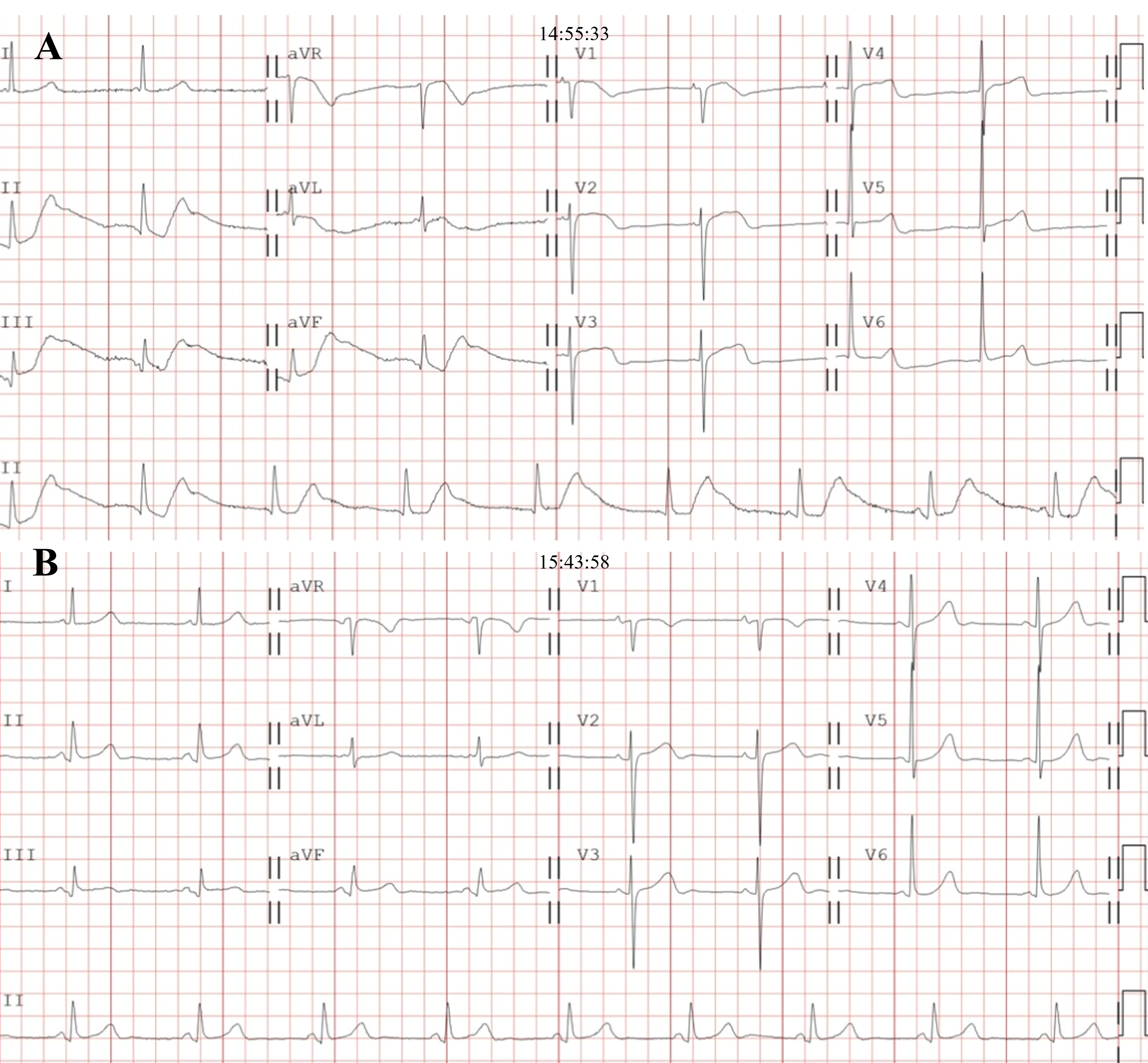

Supplement: ytag483_Supplementary_Data [file ytag483_supplementary_data.zip › S1.tif]

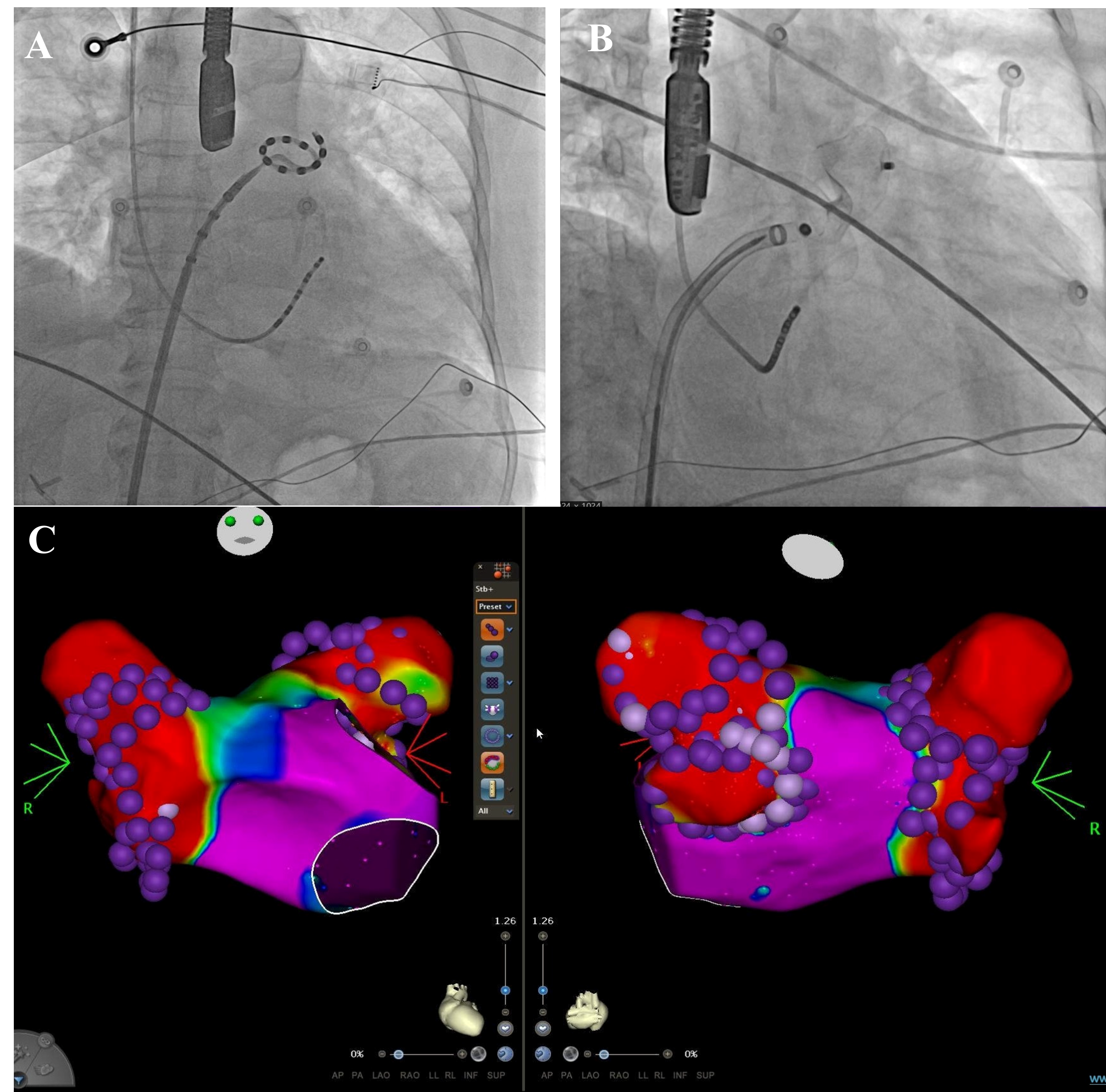

Supplement: ytag483_Supplementary_Data [file ytag483_supplementary_data.zip › S2.tif]
